# Auto-Modal: Air-Quality Index Forecasting with Modal Decomposition Attention

**DOI:** 10.3390/s22186953

**Published:** 2022-09-14

**Authors:** Yiren Guo, Tingting Zhu, Zhenye Li, Chao Ni

**Affiliations:** College of Mechanical and Electronic Engineering, Nanjing Forestry University, Nanjing 210037, China

**Keywords:** modal decomposition, air quality, short-term forecast, bidirectional encoder representation from the transformer

## Abstract

The air-quality index (AQI) is an important comprehensive evaluation index to measure the quality of air, with its value reflecting the degree of air pollution. However, it is difficult to predict the AQI accurately by the commonly used WRF-CMAQ model due to the uncertainty of the simulated meteorological field and emission inventory. In this paper, a novel Auto-Modal network with Attention Mechanism (AMAM) has been proposed to predict the hourly AQI with a structure of dual input path. The first path is based on bidirectional encoder representation from the transformer to predict the AQI with the historical measured meteorological data and pollutants. The other path is a baseline to improve the generalization ability based on predicting the AQI by the WRF-CMAQ model. Several experiments were undertaken to evaluate the performance of the proposed model, with the results showing that the auto-modal network achieves a superior performance for all prediction lengths compared to some state-of-the-art models.

## 1. Introduction

As a result of the serious environmental problems associated with industrialization and urbanization, air pollution has received a great deal of attention [1]. A report released by the World Health Organization states that almost all (99%) of the world’s population lives in an environment of air pollution, which leads to 4.2 million deaths yearly [2]. Thus, outdoor air pollution has become a serious hazard to the population. Air-quality forecasting is used to predict air pollution in advance, to provide effective guidance for protection and suppression when the air is contaminated, and to reduce the impact on health and the environment. Thus, in recent years, improved air-quality prediction model accuracy has been required.

The air-quality index (AQI) is an index that reflects the air quality. A large number of AQI prediction models, which are based on physics and chemistry or are driven by data, have been developed in the field of air-quality prediction. Although they are robust, the physics and chemistry models, e.g., the Community Multi-scale Air Quality model (CMAQ) [3], the Weather Research and Forecast model (WRF) [4], and the Nested Research and Forecasting model (NAQPMS) [5], are not sufficiently accurate. However, the commonly used WRF-CMAQ model—a combination of the WRF and CMAQ models—cannot produce optimal results due to the uncertainty inherent in the simulated meteorological field and emission inventory, and because the formation mechanism of pollutants such as ozone [6] is unclear.

With the development of computer technology, data-driven models have come to the forefront. Multi-Layer Perceptron (MLP) was designed for the prediction of sulfur dioxide concentration by Boznar et al. [7] in 1997. Since then, machine learning has been widely used in air-quality index prediction. In order to improve AQI prediction accuracy, different methods have been combined. For example, empirical modal analysis was proposed as the support vector machine input [8]. Zhao et al. [9] then proposed a temporal–spatial model combined with the k-nearest neighbor algorithm to extract meteorological data features for air-pollution grade prediction. In recent years, deep learning has been applied to air-quality prediction. Models based on deep learning methods usually achieve a higher accuracy with a much more complex structure and larger amounts of data when compared with traditional models. In 2016, the attention mechanism was introduced to attention-based RNN to search for long-term features in a time series [10], and then followed by the Long Short-Term Memory (LSTM) model to predict air quality in 2017 [11]. Ge et al. [12] used the Multi-Scale Spatiotemporal Graph Convolution Network (MST-GCN) for air-quality prediction. Compared to LSTM, the Root Mean Square Error (RMSE) of the MST-GCN was reduced by 31%; however, it is very complex. Moreover, it is difficult to obtain sufficient data for training state-of-the-art models based on deep learning [13] due to the limited number of meteorological stations in each city. Therefore, most of the published models do not meet both the accuracy and stability requirements [14].

Therefore, herein, we propose a novel Auto-Modal Attention Mechanism (AMAM) and introduce an extra additive path to the Bidirectional Encoder Representation from Transformer (BERT). In the proposed process, the transformer model with the AMAM and extra path takes measured meteorology data as the input to predict the future AQI. In particular, the proposed model requires a reference from data predicted by traditional models such as WRF-CMAQ. In this paper, the meteorology or pollutant prediction from the traditional model is referred to as first-stage predicted data, while the prediction from the proposed model is referred to as second-stage prediction.

The main contributions of this paper are as follows:

1. A novel attention mechanism, i.e., AMAM, is proposed to extract different modalities from input time-series data; from this, the decomposition weights can be automatically learned in the training process.
2. An extra additive path is introduced to collect decomposed modalities, with these values added to the first-stage prediction data.

## 2. Related Theoretical Background

### 2.1. Transformer

A transformer abandons the traditional CNN and RNN structure, i.e., the whole network structure is entirely composed of the attention mechanism. To be more precise, the transformer consists of encoder and decoder stacks. The encoder block contains two add-norm layers followed by a multi-head self-attention mechanism and feed-forward neural network, respectively. Each decoder block inserts a layer that performs attention over the output of the encoder stack based on the same structure as the encoder block [15]. The particular attention used in the transformer is known as the Scaled Dot-Product Attention Mechanism (SDPAM), which can be described by the following formula:

(1) $$\mathrm{SDPAM}(Q,K,V)=\mathrm{softmax}\left(\frac{QK^{T}}{\sqrt{d_{k}}}\right)V$$

where Q, K, and V represent the queries, keys, and values of the attention mechanism, respectively, and $d_{k}$ is the dimension of the queries and keys.

The transformer was originally designed for translation tasks. In common time-series prediction tasks, it is not as impressive as it is in the field of natural language processing [16].

### 2.2. Informer

As an improvement of the transformer in time-series forecasting, an informer is capable of predicting long-sequence time series by generating long sequential outputs through one forward procedure [17]. This improvement reduces the accumulation of prediction errors in the traditional step-by-step method. Moreover, the informer proposes an efficient self-attention mechanism, which performs scaled dot-product attention simply on dominant queries with less computational cost.

### 2.3. Bidirectional Encoder Representation from Transformer

**Figure 1:**
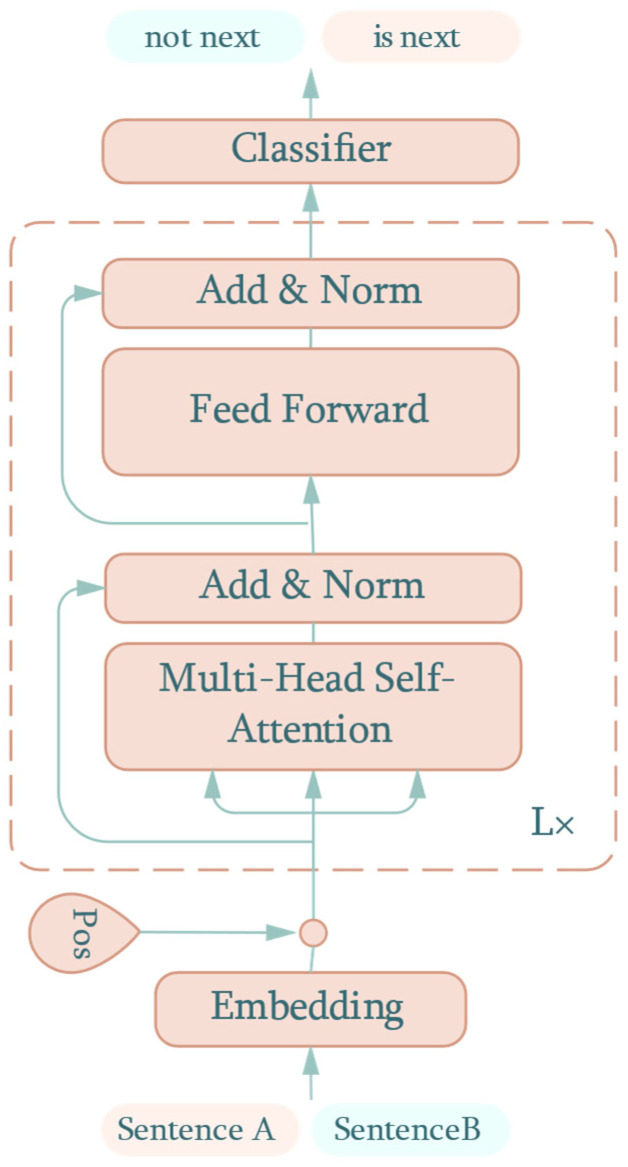
BERT structure for next sentence prediction.

Bidirectional Encoder Representation from Transformer (BERT) is a state-of-the-art method of extracting features from natural language by utilizing the encoder structure from the transformer [18]. With the idea of embedding bidirectional context information, BERT has been shown to achieve remarkable results on 11 different natural language processing tasks. A summary of the BERT structure for next sentence prediction is show in Figure 1.

The dotted box denotes the same encoder block as the transformer. The bidirectional context feature from the two input sentences flows through L numbers of consecutive encoder blocks. Finally, a classifier, such as Softmax, outputs the probability that B is the next sentence to follow A or not.

## 3. Materials and Methods

### 3.1. Data Collection

To predict air quality, a set of measured meteorology and pollutant data were collected from a meteorological station in Shanxi, China, from 23 July 2020 to 13 July 2021. Both types of data were obtained with a 1 h sampling frequency, and the pollutant data were collected with the ZR-7250 ambient air quality continuous automated monitoring system produced by Qingdao Junray Intelligent Instrument Co., Ltd., Qingdao, China. The air quality monitoring system cloud measures sulfur dioxide (SO_2_), nitrogen dioxide (NO_2_), PM_10_, PM_2.5_, ozone (O_3_), and carbon monoxide (CO), and meteorological variables such as wind speed, wind direction, etc.

To evaluate the ambient air quality quickly and accurately, systematic and effective evaluation methods have been developed, with the air-quality index (AQI) one of the indices that is widely used at present. The AQI is calculated with the above six pollutants and is a dimensionless index that quantitatively presents the air-quality status [19,20]. Therefore, the AQI was set as a comprehensive forecast target to environmental air quality in this study.

Finally, five measured meteorological variables—temperature, relative humidity, station pressure, wind speed, and wind direction—were collected, while the six pollutant concentrations above were collected. Table 1 lists the detail of samples for this work.

### 3.2. Data Preprocessing

As a result of the abnormal and null values caused by device maintenance at the meteorology station, neither the pollutant nor meteorology datasets could be directly used for the proposed model. It was necessary to fill the time series and remove the effect of outliers. In this paper, null values of measured data were repaired using the linear interpolation method and the corresponding AQI was recalculated.

The wind direction and speed of the meteorological parameters indicate air motion with great nonlinearity, and this is one of the major factors effecting the ambient air-quality trend [20]. They were replaced by the eastward component, $w_{x}$, and the northward component, $w_{y}$, of the wind speed as follows:

(2) $$\begin{array}{l} w_{x}=w_{s}\cdot \sin\left(\frac{w_{r}\cdot \pi }{180}\right)\\ w_{y}=w_{s}\cdot \cos\left(\frac{w_{r}\cdot \pi }{180}\right) \end{array}$$

where $w_{r}$ is the measured wind direction and $w_{s}$ is the measured wind speed.

**Figure 2:**
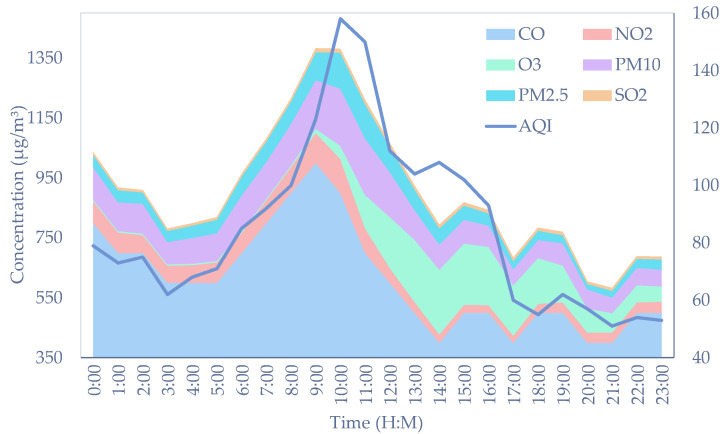
Concentration of pollutants and calculated AQI.

After processing abnormal values and converting the wind parameters, data previews were performed. As shown in Figure 2, the left axis measures the concentration of each pollutant at 1 July 2021 in the form of the stacked area and the right axis denotes the calculated AQI. More specifically, CO has the greatest stacked area, which means that it comprises the highest content of these pollutants. It can be seen that the concentration of pollutants varies considerably and CO is several orders of magnitude higher than SO_2_, which is the pollutant of minimum proportion.

It was therefore necessary to normalize the original data because they were comprised of different units and scales, which may induce vanishing or exploding gradients and poor signal propagation through the model [21]. Min-max normalization was used to rescale the sets of meteorological and pollutant data in the range of 0 to 1. The formula is as follows:

(3) $$x_{scale}=\frac{x-x_{\min}}{x_{\max}-x_{\min}}$$

where $x_{\min}$ is the minimum of the data, $x_{\max}$ is the maximum of the data, and $x_{scale}$ is the normalized result.

### 3.3. Auto-Modal Network for Predicting AQI

**Figure 3:**
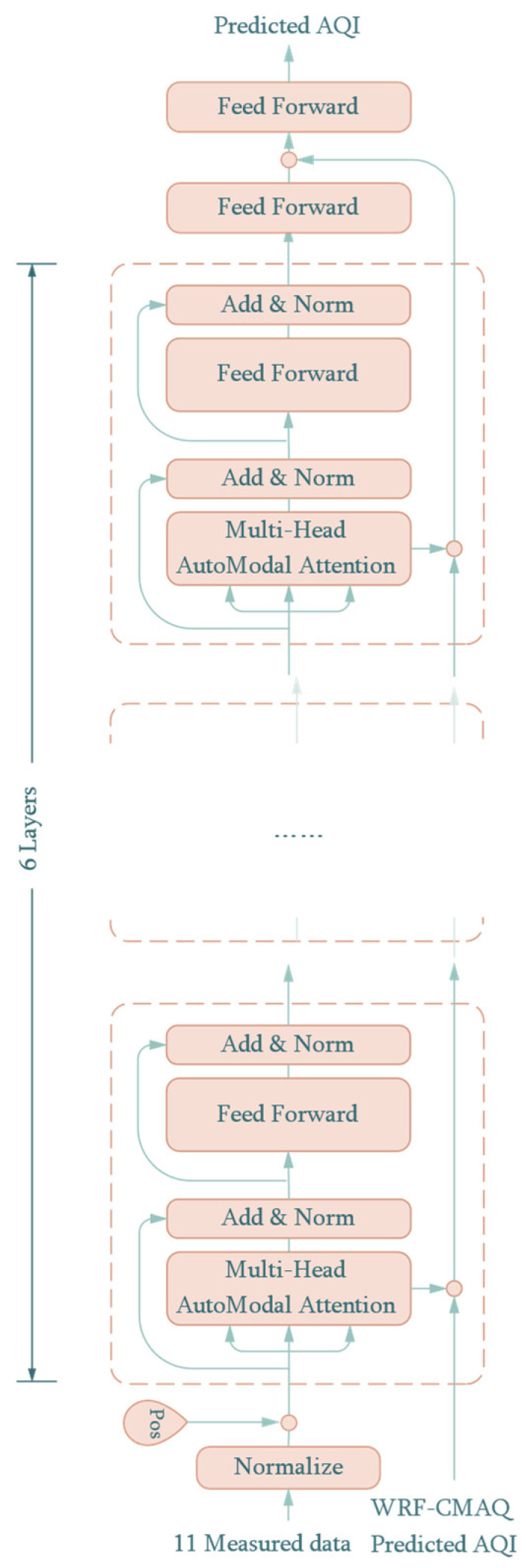
Prediction process of the auto-modal network.

On the basis of the BERT structure, we propose an end-to-end Auto-Modal network with pure encoders, which predicts the AQI directly instead of calculating the AQI on the basis of predicted airborne substances individually. Figure 3 shows the structure of the proposed model for predicting the AQI, which is a model with bidirectional time-series inputs. One of the time-series inputs of the proposed model is the historical measured meteorological parameters and pollutants which determine the AQI. The other time-series input is the AQI, which is calculated with the six pollutants used for prediction in the WRF-CMAQ model, and it is a baseline value to improve the prediction accuracy and increase the generalization ability of the proposed model.

Specifically, the historical measured meteorological variables include temperature, relative humidity, station pressure, eastward wind speed, and northward wind speed, while the six pollutant concentrations are CO, NO_2_, O_3_, PM_10_, PM_2.5_, and SO_2_. Finally, 11 measured historical variable time-series data for the previous 48 h are set as inputs of the proposed model, so that the input dimension is 11 × 48 + 1, where the second item is the predicted AQI from the first-stage prediction with the WRF-CMAQ model.

The proposed network consists of six encoder blocks with AMAM (the hidden dimension value is 48). The context from AMAM was treated as the input of the next layer, while the correction value was added to the AQI predicted by WRF-CMAQ. As a result, it was corrected six times and finally added between the two feed-forward layers in the output. This works in the same way as the residual connect, thus limiting the floor level of the prediction effect. The structure of the last two feed-forward networks is shown in Table 2. It should be noted that the first linear layer in Table 2 is used to project the input features to a shape that can perform the broadcast addition.

**Figure 4:**
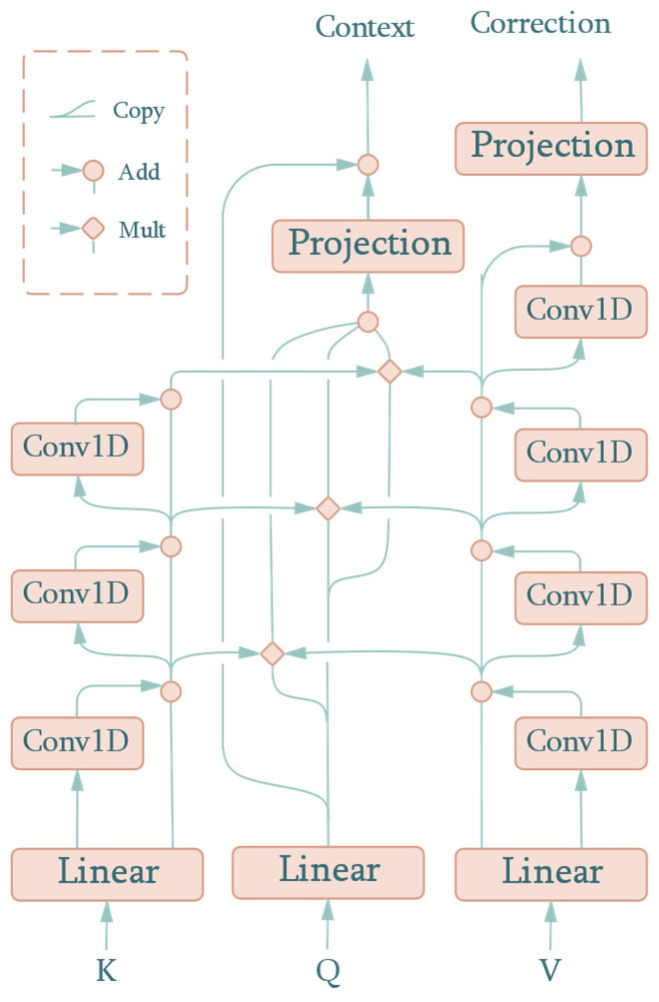
AMAM structure.

The SDPAM from the transformer directly performs the dot product on queries and keys to evaluate the distance, which represents the similarity between them. It is a reasonable method to process embedded word vectors as there is no physical meaning in neutral language. Unfortunately, SDPAM failed to achieve a similar performance with meteorological and pollutant data in the form of a time series. The meteorological and pollutant data contain a series of modals that represent hourly or daily periodic change, or even random noise caused by sensors. In order to extract inherent timing information, we propose an auto-modal scoring function, with the attention mechanism called the Auto-Modal Attention Mechanism (AMAM). The structure of AMAM is shown as Figure 4.

Similar to SDPAM, AMAM takes queries, keys, and values as inputs. However, if treated as a black box, there is a difference in the output, where AMAM adds another output, i.e., the correction value. This component represents the correction value added to the predicted AQI in the first-stage prediction. As can be seen in Figure 4, keys, queries, and values are projected to the hidden dimension, m, of the attention mechanism by a learnable linear layer:

(4) $$\begin{array}{l} K_{proj}^{l\times m}=K_{input}^{l\times n}\times W_{K}^{n\times m}\\ Q_{proj}^{l\times m}=Q_{input}^{l\times n}\times W_{Q}^{n\times m}\\ V_{proj}^{l\times m}=V_{input}^{l\times n}\times W_{V}^{n\times m} \end{array}$$

where the superscripts in the expression denote the size of the matrix and $K_{proj}^{l\times m}$, $Q_{proj}^{l\times m}$, and $V_{proj}^{l\times m}$ are the projected values that correspond to inputs $K_{input}^{l\times m}$, $Q_{input}^{l\times m}$, and $V_{input}^{l\times m}$, respectively. Finally, $W_{K}^{n\times m}$, $W_{Q}^{n\times m}$, and $W_{V}^{n\times m}$ are the weights matrices of the linear projection layer.

**Figure 5:**
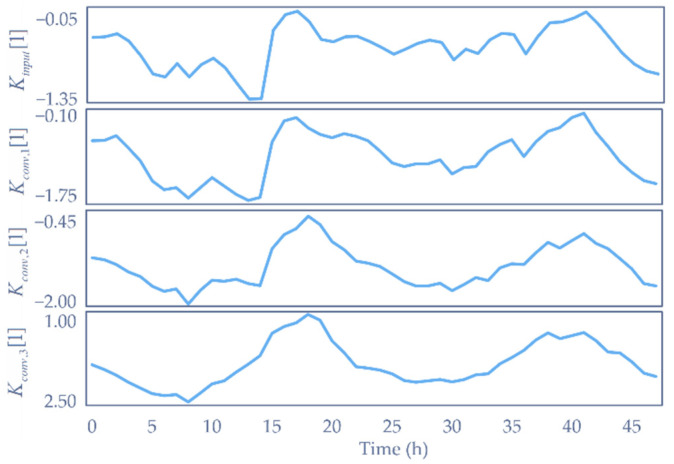
Modals of key decomposed by convolution.

Thereafter, the three one-dimensional convolutions are successively performed along the time axis over $K_{proj}$, with the residual connections acting to avoid a vanishing or exploding gradient when it becomes deep. In this paper, the kernel size of one-dimensional convolutions is 13 and the padding is 6, which makes the result the same length as the input sequence. We obtained the result after each convolution, namely, $K_{conv,1}$, $K_{conv,2}$, and $K_{conv,3}$. The expression in the forward phase is as follows:

(5) $$\left\{\begin{array}{l} K_{conv,i}^{l\times m}=\mathrm{DWConv}1d(K_{conv,i-1}^{l\times m},W_{conv,i}^{13\times m})+K_{conv,i-1}^{l\times m},i\in N^{+}\\ K_{conv,0}^{l\times m}=K_{input}^{l\times m} \end{array}\right.$$

where $W_{conv,i}^{13\times m}$ is the kernel of the ith depthwise convolution, $K_{conv,i}^{l\times m}$ is the sum of the ith depthwise convolution and its input, and $K_{input}^{l\times m}$ is the input key of the attention mechanism. In order to illustrate the effect of our network more clearly, the input and output of the key decomposition component are plotted in Figure 5. $K_{conv,i}[j]$ represents the jth column vector from $K_{conv,i}$. Therefore, the first plot is the input signal intensity along with the time sequence, with the following three plots denoting the intuited decomposed values. The *p*-values of min-max normalized $K_{conv,i}$ from an augmented Dickey–Fuller test are 0.0155, 0.0049, 0.0005, and 0.0002. They show that the deeper the time-series inputs go, the smoother and more stable the signal curve is, which means that more useful information concerned with time changing is extracted from the raw input, i.e., each $K_{conv,i}$ consists of time sequences with modals for details of different scales. All the convolutional kernels extracting these modals are learned automatically when trained. Therefore, the whole structure is named the auto-modal attention mechanism. Similarly, values are processed in the same way except that one more convolution is performed.

**Figure 6:**
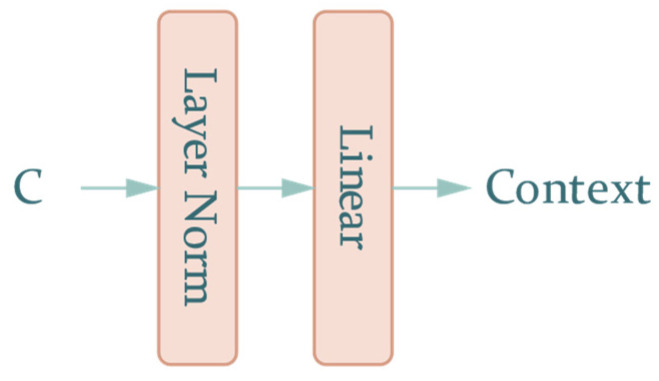
The projection network of $C^{l\times m}$.

To evaluate the similarity between keys and queries, we introduced the dot product from the transformer. Three copies of $Q_{proj}$ were made and paired with $K_{conv,1}$, $K_{conv,2}$, and $K_{conv,3}$, respectively, to reflect multi-scaled sequential patterns and boost the effective features. However, no Softmax was applied to the score before inner production with the convoluted values, $V_{conv,i}$. The reason for this is that the exponential form of Softmax may make the subsequent network structure lose sight of modals close to zero. These modals often represent high-frequency components such as random wind. From the perspective of the attention mechanism, the final value obtained by the query can be the linear combination of existing values according to their scores:

(6) $$C^{l\times m}=\sum_{i=1}^{3}(K_{conv,i}^{l\times m}⊙Q_{proj}^{l\times m})V_{conv,i}^{l\times m}$$

where $C^{l\times m}$ denotes the context value and ⊙ denotes inner production. The projection network for the path of queries is the same as that for the path of values. It consists of a feed-forward layer containing a norm and linear layer, as shown in Figure 6.

As mentioned above, context and correction can be calculated with the following formula:

(7) $$\begin{array}{l} Context=\mathrm{Norm}(C^{l\times m})\times W_{Con}^{m\times n}\\ Correction=\mathrm{Norm}(V_{conv,4}^{l\times m})\times W_{Cor}^{m\times n} \end{array}$$

where $W_{Con}^{m\times n}$ and $W_{Cor}^{m\times n}$ are the weights of the two linear layers. It should be noted that the correction output is calculated based on $V_{conv,4}^{l\times m}$ because the convolution in the key-decomposition and value-decomposition components share weights in the corresponding layers. This means that $W_{conv,i}^{13\times m}$ is the kernel of both the ith convolution of values and the keys for that input of values are exactly the keys in the self-attention, with no need to update the weights repeatedly. It is obvious that $W_{conv,4}^{13\times m}$ acts only on the value-decomposition component; thus, it is responsible for learning the feature of the correction value.

**Figure 7:**
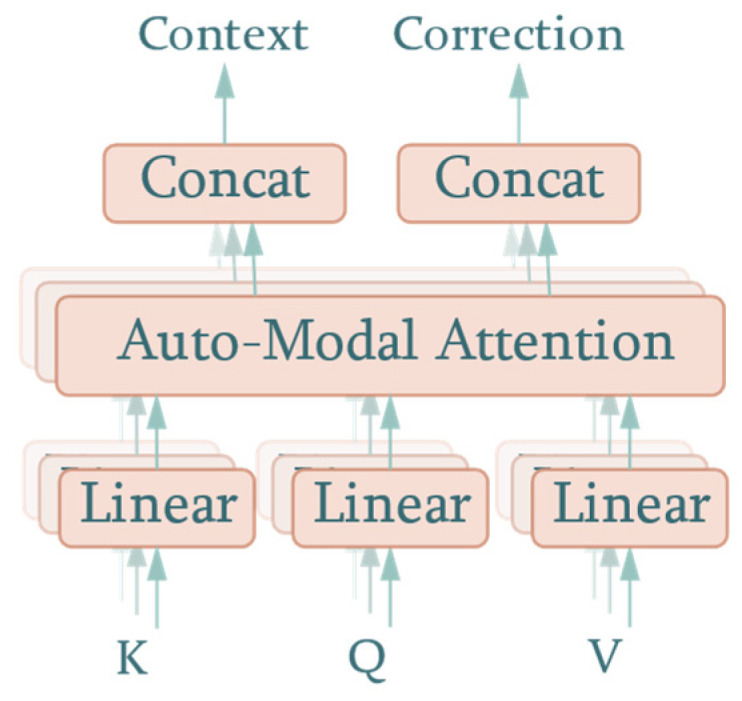
Multi-head attention with multi-output.

We also converted the multi-head structure into a multi-output compatible form by adding two separate concatenate operations to the contexts and correction values, as shown in Figure 7.

## 4. Results and Discussion

To evaluate the performance of the models, our experiments were performed on a server with Nvidia GeForce RTX 2080Ti and Intel Core I9-9900K CPU using the PyTorch 1.10 deep learning framework. In all experiments, Mean Squared Error was used as the loss function.

### 4.1. Evaluation Metrics

Normalized Mean Bias Error (nMBE), Normalized Mean Absolute Error (nMAE), Normalized Root Mean Squared Error (nRMSE), and Mean Absolute Percentage Error (MAPE) [22] were used to judge the accuracy of the predicted AQI and evaluate the performance of the models. The calculation formulae are as follows:

(8) $$\mathrm{nMBE}=\frac{1}{N}\overset{N}{\underset{i=1}{\sum }}\frac{{\hat{y}}_{i}-y_{i}}{\bar{y}}$$

(9) $$\mathrm{nMAE}=\frac{1}{N}\overset{N}{\underset{i=1}{\sum }}\frac{\left|{\hat{y}}_{i}-y_{i}\right|}{\bar{y}}$$

(10) $$\mathrm{nRMSE}=\frac{1}{\bar{y}}\sqrt{\frac{1}{N}\overset{N}{\underset{i=1}{\sum }}{\left({\hat{y}}_{i}-y_{i}\right)}^{2}}$$

(11) $$\mathrm{MAPE}=\frac{1}{N}\overset{N}{\underset{i=1}{\sum }}\left|\frac{{\hat{y}}_{i}-y_{i}}{y_{i}}\right|$$

where N is the number of all samples, ${\hat{y}}_{i}$ is the predicted value of the model, $y_{i}$ is the measured value, and $\bar{y}$ is the mean of all measured values. In addition, the persistence model is typically used as the baseline model when evaluating time-series data prediction precision [23]. The output of the persistence model is defined as:

(12) AQI(t+1)=AQI(t)

where, AQI(t) is the AQI calculated from the measured pollutants at the current moment and AQI(t+1) represents data at 8, 16, 24, 32, 40, or 48 h in the future, depending on the specific experiment. We used the Relative Forecast Power (*RFP*) to assess the performance related to a baseline model:

(13) $$\begin{array}{l} RFP=\mathrm{sgn}(-E_{\mathrm{nRMSE}})\sqrt{\left|\frac{E_{\mathrm{nRMSE}}E_{\mathrm{MAPE}}}{{\mathrm{MAPE}}_{b}\times {\mathrm{nRMSE}}_{b}}\right|}\times 100\%\\ E_{\mathrm{nRMSE}}={\mathrm{nRMSE}}_{f}-{\mathrm{nRMSE}}_{b}\\ E_{\mathrm{MAPE}}={\mathrm{MAPE}}_{f}-{\mathrm{MAPE}}_{b} \end{array}$$

where nRMSE*_f_* and nRMSE*_b_* are the nRMSE of the evaluated model and baseline model, respectively, and MAPE*_f_* and MAPE*_b_* are the MAPE of the evaluated model and baseline model, respectively.

### 4.2. Performance of Auto-Modal Network

In this section, we compare our model with two commonly used models in the fields of Natural Language Processing (NLP). One of them is an RNN-based model, i.e., LSTM. Another model is the transformer, the foundation of various state-of-the-art NLP methods. To compare the performances under different horizon scales, we selected a wide range of prediction lengths, from 8 h to 48 h: L∈{8,16,24,32,40,48}. All the models were trained with MSE loss using the SGD optimizer at a learning rate of 10^−4^ and were not interrupted until convergence. The batch size was set to 1024.

In Table 3, the best entries are in bold. The auto-modal network exhibited the superior performance in the experiment. Considering the average value of each metric along different prediction lengths, LSTM and the transformer, respectively, were 11.5% and 4.2% behind for nRMSE, 14.0% and 6.1% for nMAE, 13.0% and 4.1% for MAPE, and, most distinctively, 321.6% and 278.4% for nMBE. This means that the auto-modal network achieved a superior performance for all prediction lengths.

### 4.3. Performance of Prediction Models

**Figure 8:**
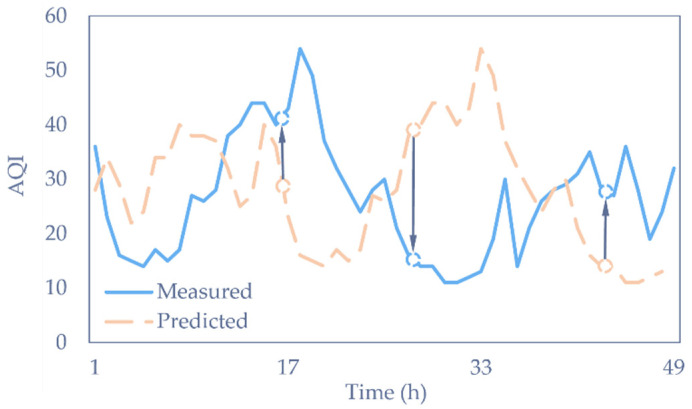
Prediction of the persistence model for 48 h.

Apart from the commonly used NLP models in Table 3, we also compared the performance of the proposed model with the other four time-series prediction models, including the baseline of the persistence model, the Temporal Convolutional Network (TCN), the informer, and the first-stage model (WRF-CMAQ). The results are listed in Table 4. A lower metric indicates a better prediction, with the best results in bold. It is obvious that the proposed model returned the best results for nRMSE, nMAE, and MAPE. The results show that the auto-modal network achieved a superior performance in all prediction lengths. However, the persistence model with poor prediction accuracy achieved the best nMBE. This can be intuitively explained by Figure 8. As illustrated in the plot, the predicted AQI had a fixed time lag after the ground truth value. Consequently, it caused the nMBE error to produce similar components with different signs. These offset each other in the calculation of the nMBE. Statistically, the AQI value is subject to Gaussian distribution, which has a stable mean value, leading the nMBE value of the persistence model to tend to zero when the quantity of samples is large enough, according to the large number theorem.

As shown in Table 4, another noticeable point is that the WRF-CMAQ model was far worse than the other models. This was because the formation mechanism of O3 was not clear, causing the physics-based or chemistry-based models to be unable to predict accurately. In the experiment, we used WRF-CMAQ to predict the concentrations of pollutants, including the poorly forecasted O3, and calculate the AQI based on these concentrations. In other words, WRF-CMAQ was not suitable to forecast indices formed of several components, such as the AQI. However, the auto-modal network was trained to make the best of the pollutants predicted in the first stage and extract their relationship instead of following the unreliable AQI.

The state-of-the-art informer model took second position in the comparison. As regards the advantages in prediction accuracy, the auto-modal network produced a 2.71% nRMSE reduction, a 4.51% nMAE reduction, and a 2.05% MAPE reduction. In particular, the proposed model produced a 119.15% reduction for nMBE, meaning that the overall deviation was much lower than that of the informer, and the prediction distribution was closer to the ground truth. This indicates that a stable projection function for historical meteorology and pollutant data was achieved, and the first-stage predicted AQI to the AQI in the future was learned.

Table 5 is the RFP of the informer and auto-modal network. The higher the RFP index, the more accurate the prediction. The auto-modal network produced an average increase of 0.83% compared with the informer in short-term predictions of 8, 16, and 24 h, and an increase of 1.05% in long-term predictions of 32, 40, and 48 h. Putting aside the accuracy of the metrics, the auto-modal network drew with the informer in terms of the stability of the long sequence prediction over the range of {8,16,24,32,40,48}. In particular, the extended prediction accuracy slightly increased the informer’s superiority with the benefit of WRF-CMAQ’s consistency.

## 5. Conclusions

An auto-modal network was developed to predict the AQI based on a novel auto-modal attention mechanism and bidirectional encoder representation from the transformer. The auto-modal network could extract different modalities from input time-series meteorological variables and pollutants to predict the AQI for different prediction lengths with a reference, a predicted AQI by the WRF-CMAQ model, in an additive path to ensure its generalization performance.

Several experiments were undertaken to evaluate the performance of the proposed auto-modal network to compare with the LSTM, TCN, transformer, informer, and persistence models for a range of prediction lengths from 8 to 48 h. The results show that the auto-modal network performed best on the evaluating indicator of nRMSE, followed by the informer model. The relative forecast powers of the proposed model all exceed 50% in different prediction lengths, with a maximum of 68.5%. The precise AQI predictions are beneficial to public health and provide guidance for pollution prevention and control.

In this work, we only focused on the AQI prediction instead of a specific pollutant. In future, we will attempt to determine the key meteorological and environmental factors for each pollutant, and then adjust the proposed model to predict air pollutants.

## Figures and Tables

**Table 1:** Details of training, validation, and test datasets.

Datasets	Number of Sample Couples	Ratio
Training	6720	80%
Validation	840	10%
Test	840	10%
Total	8400	100%

**Table 2:** Structure of the two feed-forward networks.

Type	Neurons/Axes/Ratio	Input Size	Position
Linear	1	48 × 48	Feed-Forward 1
Transpose	1, 2 ^1^	48 × 1	Feed-Forward 1
Broadcast Add	-	1 × 1, 1 × 48 ^2^	-
Linear	128	1 × 48	Feed-Forward 2
Dropout + ReLU	0.4	1 × 128	Feed-Forward 2
Linear	32	1 × 128	Feed-Forward 2
Dropout + ReLU	0.4	1 × 32	Feed-Forward 2
Linear	16	1 × 32	Feed-Forward 2
Dropout + ReLU	0.4	1 × 16	Feed-Forward 2
Linear	1	1 × 16	Feed-Forward 2

^1^ Axes 1 and 2 are to swap. ^2^ Input of 1 × 1 is repeated 48 times to the shape of 1 × 48 for addition operation.

**Table 3:** Metrics of the auto-modal and NLP models with different prediction lengths.

Models	Metrics	Prediction Length (Hours)						
		8	16	24	32	40	48	Mean ^1^
Auto-Modal	nRMSE	**0.0363**	**0.0378**	**0.0379**	**0.0439**	**0.0308**	**0.0321**	**0.0365**
	nMBE	**−0.0099**	0.0162	**0.0032**	**0.0054**	0.0168	**−0.0014**	**0.0051**
	nMAE	**0.1221**	**0.1086**	**0.1057**	**0.1121**	0.1100	**0.1124**	**0.1118**
	MAPE	**0.1425**	**0.1299**	**0.1286**	**0.1326**	**0.1351**	**0.1332**	**0.1337**
LSTM	nRMSE	0.0382	0.0398	0.0430	0.0543	0.0344	0.0345	0.0407
	nMBE	−0.0385	**−0.0074**	−0.0323	−0.0241	−0.0150	−0.0118	−0.0215
	nMAE	0.1360	0.1173	0.1186	0.1479	0.1219	0.1231	0.1275
	MAPE	0.1540	0.1404	0.1380	0.1782	0.1475	0.1485	0.1511
Transformer	nRMSE	0.0390	0.0396	0.0396	0.0452	0.0313	0.0336	0.0381
	nMBE	−0.0311	−0.0201	−0.0178	−0.0125	**−0.0100**	−0.0241	−0.0193
	nMAE	0.1372	0.1151	0.1107	0.1161	**0.1098**	0.1226	0.1186
	MAPE	0.1573	0.1342	0.1328	0.1348	0.1359	0.1403	0.1392

^1^ Average value of the metrics of prediction lengths of 8, 16, 24, 32, 40, and 48 h.

**Table 4:** Metrics of the auto-modal and prediction models with different prediction lengths.

Models	Metrics	Prediction Length (Hours)						
		8	16	24	32	40	48	Mean ^1^
Auto-Modal	nRMSE	**0.0363**	**0.0378**	**0.0379**	**0.0439**	**0.0308**	**0.0321**	**0.0365**
	nMBE	−0.0099	0.0162	0.0032	0.0054	0.0168	**−0.0014**	0.0051
	nMAE	**0.1221**	**0.1086**	**0.1057**	**0.1121**	**0.1100**	**0.1124**	**0.1118**
	MAPE	**0.1425**	**0.1299**	0.1286	**0.1326**	**0.1351**	**0.1332**	**0.1337**
TCN	nRMSE	0.0416	0.0499	0.0553	0.0496	0.0386	0.0364	0.0452
	nMBE	−0.0034	−0.0028	−0.0269	−0.0247	−0.0101	−0.0243	−0.0154
	nMAE	0.1452	0.1512	0.1559	0.1318	0.1382	0.1330	0.1426
	MAPE	0.1694	0.1848	0.1866	0.1583	0.1702	0.1593	0.1714
WRF-CMAQ	nRMSE	0.1201	0.1388	0.1455	0.1507	0.1132	0.1122	0.1301
	nMBE	−0.1606	−0.1565	−0.1722	−0.1689	−0.1844	−0.1676	−0.1684
	nMAE	0.4389	0.4180	0.4136	0.4216	0.4184	0.4176	0.4214
	MAPE	0.4433	0.4213	0.4130	0.4229	0.4178	0.4132	0.4219
Informer	nRMSE	0.0371	0.0392	0.0383	0.0457	0.0316	0.0331	0.0375
	nMBE	−0.0262	−0.0249	−0.0230	−0.0306	−0.0302	−0.0237	−0.0264
	nMAE	0.1298	0.1132	0.1082	0.1174	0.1148	0.1191	0.1171
	MAPE	0.1513	0.1299	**0.1261**	0.1357	0.1382	0.1374	0.1365
Persistence	nRMSE	0.0805	0.0892	0.0882	0.0967	0.0969	0.0959	0.0912
	nMBE	**0.0001**	**−0.0002**	**−0.0006**	**−0.0005**	**−0.0010**	−0.0016	**−0.0006**
	nMAE	0.2908	0.3200	0.3018	0.3529	0.3576	0.3425	0.3276
	MAPE	0.3418	0.3766	0.3458	0.4210	0.4331	0.4063	0.3874

^1^ Average value of the metrics of prediction lengths of 8, 16, 24, 32, 40, and 48 h.

**Table 5:** RFP of the informer and auto-modal network.

Models	Prediction Length (Hours)						
	8	16	24	32	40	48	Mean ^1^
Auto-Modal	56.57%	61.47%	59.83%	61.16%	68.50%	66.85%	62.70%
Informer	54.80%	60.60%	59.96%	59.80%	67.74%	65.82%	61.77%

^1^ Calculated with the average value of the metrics in Table 3.
